# Analysis of Volatile Compounds’ Changes in Rice Grain at Different Ripening Stages via HS-SPME-GC–MS

**DOI:** 10.3390/foods13233776

**Published:** 2024-11-25

**Authors:** Liting Zhang, Zhaoyang Pan, Zhanhua Lu, Shiguang Wang, Wei Liu, Xiaofei Wang, Haoxiang Wu, Hao Chen, Tengkui Chen, Juan Hu, Xiuying He

**Affiliations:** 1 Guangdong Rice Engineering Laboratory, Guangdong Key Laboratory of Rice Science and Technology, Key Laboratory of Genetics and Breeding of High Quality Rice in Southern China (Co-Construction by Ministry and Province), Ministry of Agriculture and Rural Affairs, Rice Research Institute, Guangdong Academy of Agricultural Sciences, Guangzhou 510640, China; zhliting1018@163.com (L.Z.); 15238535392@163.com (Z.P.); luzhanhua@gdaas.cn (Z.L.);; 2 Binhai College of Agriculture, Guangdong Ocean University, Zhanjiang 524088, China

**Keywords:** aromatic rice, HS-SPME-GC-MS, VOCs, rOAV

## Abstract

Aroma is a crucial determinant of rice taste quality, with volatile organic compounds (VOCs) playing a key role in defining this characteristic. However, limited research has explored the dynamic changes in these aromatic substances during the ripening stages of rice grains. In this study, we analyzed VOCs in rice grains across four ripening stages post-flowering using headspace solid-phase microextraction combined with gas chromatography–mass spectrometry (HS-SPME-GC-MS). A total of 417 VOCs were identified, among which 65 were determined to be key aroma-active compounds based on relative odor activity value (rOAV) analysis. Most of these aroma-active compounds exhibited an accumulation pattern as the grains matured. Notably, 5-ethyl-3-hydroxy-4-methyl-2(5H)-furanone and 2-Methyloxolan-3-one had the largest rOAV values. Additionally, (Z)-6-nonenal, (Z,Z)-3,6-nonadienal, 2-thiophenemethanethiol, 5-methyl-2-furanmethanethiol, 2,2,6-trimethyl-cyclohexanone, and 3-octen-2-one were identified as potential key markers for distinguishing rice-grain maturity stages. Moreover, 2-acetyl-1-pyrroline (2-AP), heptanal, and 1-nonanol were identified as marker metabolites differentiating aromatic from non-aromatic brown rice. These findings contribute to a deeper understanding of the dynamic variation and retention of aroma compounds during rice-grain ripening, and they offer valuable insights into the improvement of fragrant rice varieties.

## 1. Introduction

Rice (*Oryza sativa* L.) is one of the world’s most important crops, and it serves as a staple food, particularly in Asia and India. In recent years, consumer preferences have increasingly focused on the quality of rice, not only in terms of its nutritional value but also its aromatic qualities. Rice produces a distinctive aroma during its growth, processing, and cooking stages, which plays a crucial role in its appeal. Due to their superior taste and aroma, aromatic rice varieties are highly sought after, and they command higher prices in the market compared to non-aromatic varieties.

The distinctive aroma of rice is primarily due to its volatile organic compounds (VOCs). These include a diverse group of chemicals such as aldehydes, ketones, organic acids, alcohols, esters, hydrocarbons, phenols, pyrazines, and pyridines, originating mainly from the metabolism of fatty acids, amino acids, and carbohydrates [1,2]. Previous research has revealed that more than 500 VOCs have been isolated from rice, and the VOCs in aromatic rice were more complex than those in non-aromatic rice [3,4]. 2-Acetyl-1-pyrroline (2-AP), was considered the key aroma constituent in scented rice, with an odor threshold of 0.1 ng g^−1^ in water and 0.02–0.04 ng L^−1^ in air [5]. However, other VOCs also have irreplaceable effects on the aroma. For instance, Setyaningsih et al. [6] found that pentanal, hexanal, 2-pentyl-furan, 2,4-nonadienal, pyridine, 1-octen-3-ol, and (E)-2-octenal are responsible for the differences between aromatic and non-aromatic rice varieties. Similarly, Yuan et al. [7] identified 2-heptanone and 2-nonanone as novel aromatic biomarkers contributing to rice aroma. In varieties lacking 2-AP, other compounds like pentan-1-ol, (E)-2-octenal, and 2-octanone contribute to a similar popcorn-like flavor [8]. Most research has focused on the aromatic profiles of raw, cooked, and stored rice at present [9,10,11,12]. As rice matures, VOCs are synthesized, transported, and accumulated in the grain. It is necessary to understand the accumulation of VOCs in rice grains at different ripening stages.

Sample extraction and final detection (gas chromatography-coupled) are two im-portant steps involved in detecting and identifying VOCs. The current standard sample extraction techniques for the analysis of VOCs are dynamic headspace (DHS), headspace solid-phase microextraction (HS-SPME), dispersive liquid–liquid microextraction (DLLME), stir-bar sorptive extraction (SBSE), and solid-phase extraction (SPE) [13]. HS-SPME is founded upon the equilibrium between the sample matrix, the headspace of the vial, and SPME fiber; it permits direct sampling and does not necessitate the use of organic solvents. Volatiles diffuse from the sample into the headspace, where they then adsorb onto the fiber and finally thermally desorb at the injection port of the GC for analysis [2,14]. The HS-SPME method is capable of extracting a greater quantity of low-molecular-weight volatiles [15]. GC-MS has been proven to be very sensitive with respect to the known and unknown VOCs, GC for detection and separation, and mass spectrometry (MS) for the identification and characterization of individual atomic compounds in the sample matrix [3]. HS-SPME is often combined with GC-MS for the identification and quantification of VOCs. HS-SPME-GC–MS is extensively utilized to analyze a diverse array of products, including rice [16,17], tea [18], passion fruit [19], prickly ash [20], pomegranate [21], walnut [22], jasminum sambac [23], and so on. Headspace gas chromatography–ion mobility spectrometry (HS-GC-IMS) combines GC and IMS to obtain VOC profiles based on the difference in the drift time of ionized VOC in a drift tube under atmospheric pressure; it is suitable for the detection of trace VOCs, and it is used to analyze the VOCs of aromatic and non-aromatic rice cultivars [24,25]. HS-SPME combined with two-dimensional gas chromatography, as well as time-of-flight mass spectrometry (GC × GC-TOFMS), can completely separate and collect spectral data in a full scan, which increases the possibility of identifying new compounds and mining potential key-marker volatile compounds. The method has previously been applied in the study of key-marker volatile compounds in aromatic rice grains [6].

The odor activity value (or relative odor activity value (rOAV)) is the ratio of the concentration of each VOC to the odor threshold (OT), serving as a critical metric for assessing the impact of specific aromatics [26]. It is generally believed that VOCs with OAV/rOAV > 1 significantly contribute to the overall aroma profile of a sample [27]. Zhao et al. [28] identified seven aroma-active compounds, including hexanal, octanal, nonanal, (E)-2-octenal, decanal, 1-heptanol, and 1-octanol in jasmine rice. Ye et al. [29] studied the changes in aromatic substances during the growth of navel oranges and indicated that 14 substances were the key aroma components, among which linalool, β-myrcene, and limonene achieved the highest OAV. Xi et al. [22] found that walnut oil odor in the early ripening stage derived from 1,8-cineole (OAV 280) and ethanol (OAV 134.5) and in the mid-ripening stage derived from nonanal (OAV 181.82), (E)-2-octenol (OAV 160), and hexanal (OAV 103.78), as well as the flavor of later ripening stage, were affected by nonanal (OAV 192.28), 1-heptanol (OAV 150), heptanal (OAV 71.11), and some organic acids. Combining the results of differential metabolites with OAV analysis allows the aroma profile of rice to be characterized, while key aromatic compounds can also be revealed.

In this study, rice grains from three different varieties were collected at four critical ripening stages: 10 days after flowering (10 d), 15 days after flowering (15 d), 25 days after flowering (25 d), and 35 days after flowering (35 d), which are the important ripening stages of rice grain. We employed HS-SPME-GC–MS to detect the VOC components of rice-grain samples at each ripening stage. Next, we explored the characteristic VOCs and differential metabolites during the ripening of rice grains using chemometrics methods. Then, we assessed the impact of these VOCs on the overall aroma profile of the rice using rOAV. This research provides insights into the evolution of aroma profiles in rice grains, providing a foundation for the quality control of aromatic rice.

## 2. Materials and Methods

### 2.1. Rice Materials

In this study, three rice cultivars were selected based on their brown rice, aromatic rice cultivars Yuexiang 430 (YX430) and Meixiangzhan No. 2 (MXZ2), as well as non-aromatic rice cultivar Yuejingsimiao No. 2 (YJSM2). All rice samples were cultivated during the same season under identical conditions at the Baiyun Test Base, Guangdong Academy of Agricultural Sciences, Guangdong Province, China. Rice-grain samples were collected at four distinct ripening stages: 10 days, 15 days, 25 days, and 35 days after flowering. The samples were harvested between 16:00 and 18:00 and immediately placed in liquid nitrogen. After collection, the rice husks were removed using tweezers while the samples were kept in liquid nitrogen and then stored at −80 °C until further use. For the subsequent metabolomic analysis, three biological replicates were prepared, each consisting of a pooled sample from at least three plants.

### 2.2. Reagents

3-Hexanone-2,2,4,4-d4 (CAS: 24588-54-3, chromatographic grade) was used as the internal standard. 3-Hexanone-2,2,4,4-d4 and N-alkanes (C7-C40, chromatographic grade) were purchased from Sigma-Aldrich (St. Louis, MO, USA). NaCl (analytical-grade) was purchased from Sinopharm Chemical Reagent Co. Ltd. (Beijing, China).

### 2.3. Sample Preparation and Treatment

The rice samples were pulverized into a fine powder using liquid nitrogen to preserve volatile components. Directly after pulverization, 0.2 g of this powder was placed into a 20 mL headspace vial (Agilent, Palo Alto, CA, USA), along with 0.2 g of NaCl powder. To this mixture, we added 20 μL (10 μg/mL) of internal standard solution (dissolved in a n-hexane solution). Each vial was immediately sealed with crimp-top caps that included TFE-silicone headspace septa (Agilent) to prevent any loss of volatiles, ensuring consistent sampling conditions.

### 2.4. HS-SPME Conditions

For the SPME analysis, each vial was pre-heated at 60 °C for 5 min. Following this, a 120 μm DVB/CWR/PDMS fiber (SPME Arrow, Agilent) was introduced to the headspace of the sample for 15 min at 60 °C in order to extract the VOCs. Prior to sampling, the fiber was conditioned at a fiber conditioning station at 250 °C for 5 min.

### 2.5. GC-MS Conditions

After sampling, the desorption of the VOCs from the fiber coating was carried out in the injection port of the GC apparatus (Model 8890; Agilent) at 250 °C for 5 min in the splitless mode. The identification and quantification of VOCs were carried out using an Agilent Model 8890 GC and a 7000 E mass spectrometer (Agilent), equipped with a 30 m × 0.25 mm × 0.25 µm DB-5MS (5% phenyl-polymethyl siloxane) capillary column (Agilent J & W Scientific, Folsom, CA, USA). Helium (purity is not less than 99.999%) was used as the carrier gas at a linear velocity of 1.2 mL/min. The injector temperature was kept at 250 °C. The oven temperature was programmed from 40 °C (3.5 min), increasing by 10 °C/min to 100 °C, by 7 °C/min to 180 °C, and by 25 °C/min to 280 °C and then held for 5 min. Mass spectra (MS) were recorded in the electron impact (EI) ionization mode at 70 eV. The quadrupole mass detector, ion source, and transfer line temperatures were set, respectively, to 150, 230, and 280 °C. The MS was used in the ion monitoring (SIM) mode to identify and quantify the analytes.

### 2.6. Qualitative and Relative Quantitative Analysis of VOC Components

The downstream raw data were processed using Mass Hunter software from Agilent Qualitative Systems following the completion of the mass spectrometry analysis. The deconvolution parameter peak width was set to 20, and the resolution, sensitivity, and chromatographic peak shape requirements were set to medium. The minimum value of the matching factor was set to 70. The identification of substances was carried out using mass spectrometry data, together with the mass spectra of NIST (2020) Spectral Library (https://sciencesolutions.wiley.com/wiley-releases-the-wiley-registry-12th-edition-nist-2020-mass-spectral-library-with-over-2-million-ei-and-lc-ms-spectra/, accessed on 15 January 2023) standards. Metabolites were qualitatively and quantitatively analyzed via mass spectrometry using a self-constructed MWGC database. The mass spectra of the volatile metabolites in the samples were obtained and subsequently analyzed by integrating peak areas. The quantitative ions were selected for integration and correction. The qualitative method used in this study was supported by the existing methodological literature [7,30].

The relative content of individual volatile components was calculated from the peak area. Semi-quantification of VOCs used the linear relationship between peak areas and concentrations of the internal standard (3-Hexanone-2,2,4,4-d4, 0.1 mg/mL) and VOCs. According to the literature calculation method [31], the relative content of VOCs in the sample was calculated according to the following formula:

$$X_{i}=\frac{V_{s}\times C_{s}}{M}\times \frac{I_{i}}{I_{s}}\times 10^{-3}$$

where *X_i_* indicates the content of the unknown component *i* (μg/g), *V_s_* signifies the injection volume of the internal standard (L), *C_s_* represents the mass concentration of the internal standard (ug/mL), *I_i_* is the peak area of the unknown component i, *I_s_* is the peak area of the internal standard, and *M* denotes the sample mass (g).

### 2.7. Calculation of rOAV

The rOAV analysis was conducted following the methods described by Huang et al. (2022) [32]. The rOAV was calculated as follows:

$$rOAV=\frac{C_{i}}{T_{i}}$$

where rOAV indicates the relative odor activity value of the components, *C_i_* signifies the relative content of the components (μg/g), and *T_i_* indicates the OT of the components (μg/g).

### 2.8. Statistical Analysis

The raw spectra obtained from the GC-MS analysis were processed utilizing the MassHunter software. For statistical evaluation, we applied a one-way ANOVA, complemented by Tukey’s post hoc test for pairwise comparisons, using SPSS version 26. Unsupervised principal component analysis (PCA) was performed using the statistics function prcomp within R (www.r-project.org, accessed on 20 December 2023). Orthogonal partial least squares–discriminant analysis (OPLS-DA) was performed using the R package MetaboAnalystR. Variable importance of projection (VIP) values, score plots, and permutation plots were extracted from the OPLS-DA results. Prior to OPLS-DA, the data were log-transformed (log2) and mean-centered. A permutation test involving 200 repetitions was implemented to safeguard against overfitting.

## 3. Results and Discussion

### 3.1. Analysis of VOC Components During the Ripening of Rice Grains

**Figure 1:**
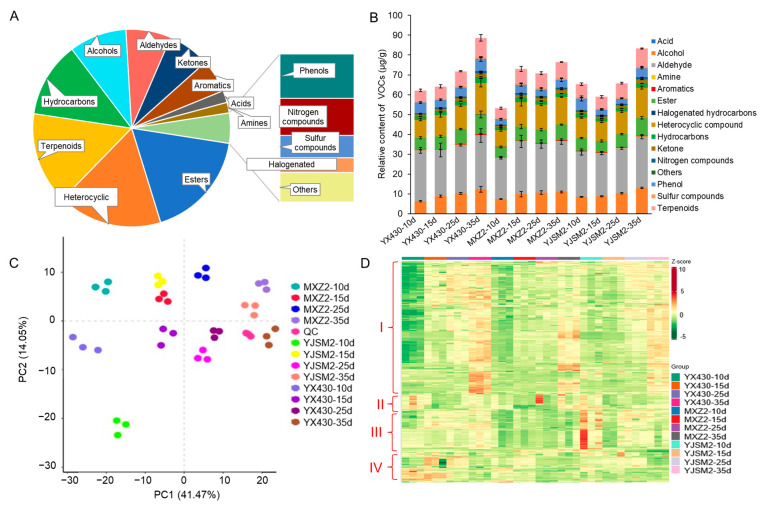
Volatile compound detection and analysis from all rice samples of YX430, MXZ2, and YJSM2 at four stages. (**A**) Classification of the 417 VOCs of 12 rice-grain samples. (**B**) The relative amounts of VOCs from YX430, MXZ2, and YJSM2 of four stages. (**C**) PCA score plot of different stages using all volatile compounds. Three same-color dots represent three repetitions of each variety in the same period. (**D**) Cluster heatmap analysis of volatile compounds of all samples. Each sample is represented as a column, and each metabolite is displayed in a row. Red shows relatively high metabolite abundance, while green indicates relatively low abundance. I, II, III and IV refer to the Group I, Group II, Group III, and Group IV, respectively.

The VOC profiles of rice-grain samples at different stages of ripening were analyzed using HS-SPME-GC-MS, with the total ion chromatogram presented in Figure S1. Across three rice cultivars at four stages post-flowering, 417 VOCs were identified in brown rice (Table S1). These VOCs were divided into esters (17.51%), heterocyclic compounds (17.03%), terpenoids (14.87%), hydrocarbons (11.99%), alcohols (9.35%), aldehydes (7.76%), ketones (7.19%), aromatics (5.28%), acids (1.92%), amines (1.92%), phenols (1.92%), nitrogen compounds (1.20%), sulfur compounds (0.72%), halogenated hydrocarbons (0.48%), and other compounds (0.96%) (Figure 1A). The total VOC content increased progressively as the rice grains matured, with the relative contents for YX430, MXZ2, and YJSM2 at the four stages being 53.18–62.15 μg/g (10 DAF), 58.96–72.99 μg/g (15 DAF), 65.74–71.78 μg/g (20 DAF), and 76.46–88.60 μg/g (35 DAF), respectively (Figure 1B). This indicates a general accumulation of VOCs as the rice grains matured.

Furthermore, we investigated the content of VOCs in different classes. Among these volatiles, aldehyde generally had high contents in all rice samples. Aldehydes are mainly produced via lipid oxidation and decomposition as the most important volatile components in raw rice because of their relatively low odor threshold [2]. These substances contribute fresh and fruity aromas to rice grains. 2,6-Dimethyl-5-heptenal and hexanal had the highest content of aldehydes and existed in the whole ripening stages. Hexanal, with a green grass-like aroma, can be used as a marker for green/grassy aromatics [33]. The levels of hexanal indicate the freshness of tested rice cultivars [24]. In stored rice, hexanal is formed through the automatic oxidation of linoleic acid resulting in an off and stale flavor [16]. What followed were heterocyclic compounds, alcohol, terpenoids, ester, and phenol. Heterocyclic compounds were the major volatile flavor compounds produced through Maillard reactions, and they were also the second most abundant substance in the study. Furans were the heterocyclic compounds, produced from both lipid oxidation and the Maillard reaction of amino acids and sugars at high temperatures [2]. The extraction temperature of VOCs from the sample was 60 °C in this study; the Maillard reaction cannot be avoided during the extraction process. So, part of furans may come from the Maillard reaction. 2-Methylfuran, 2-pentylfuran, etc. play important roles in forming rice aroma [2]. 2-Pentylfuran has a characteristic nutty odor in dilute concentration; it was reported as an odor-active compound in aromatic, non-aromatic, and black rice [4,34]. Alcohols were formed from the further breakdown of aldehydes; they made some contribution to the overall aroma of rice, and the content was second only to heterocyclic compounds. Terpenoids exist widely in plants. Almost all flower-aroma volatile components contain terpenoids, which are also characteristic aroma components of some fruits [35]. In contrast, hydrocarbons and ketones were present at lower levels, ranging from 1 μg/g to 3 μg/g across all samples. Additionally, the contents of aromatics, acids, amines, nitrogen compounds, sulfur compounds, and halogenated hydrocarbons were minimal, each with concentrations below 1 μg/g in all rice samples. Overall, the components of VOCs in aromatic and non-aromatic rice were the same, except for some special substances. The volatile compounds in aromatic rice (YX430 and MXZ2) were more abundant than those in non-aromatic rice (YJSM2).

### 3.2. Chemometric Analysis of VOCs

PCA is a widely employed technique for analyzing multidimensional data sets in an unsupervised fashion. The PCA result can show the trend of metabolome separation between the groups and indicate the differences in the metabolome within the sample group [36,37]. To better distinguish and present the differences in VOCs in rice grains across different ripening stages, we conducted a PCA on all samples based on their VOC profiles. The PCA results showed that component 1 (PC1) and component 2 (PC2) explained 41.47% and 14.05% of the variance across the four ripening stages in three rice cultivars, respectively (Figure 1C). PCA revealed a lower variability among the biological replicates. The rice grain of four ripening stages was obviously separated. The separation of all samples along PC1 and PC2 underscores the significant metabolic diversity among the different ripening stages.

Additionally, a hierarchical clustering analysis (HCA) was performed on all detected VOCs, with the results presented as a heatmap (Figure 1D). The metabolite accumulation patterns of the four periods were different. The VOCs were categorized into four groups: Group I, Group II, Group III, and Group IV. The relative content of VOCs in Group I and Group III gradually increased as the grains matured, while the content in Group II and Group IV progressively decreased. The content of VOCs in rice grains undergoes a dynamic shift during ripening, with certain VOCs increasing or decreasing as the grains mature and others peaking at specific stages. This accumulation pattern caused by grain maturation was further supported by HCA.

**Figure 2:**
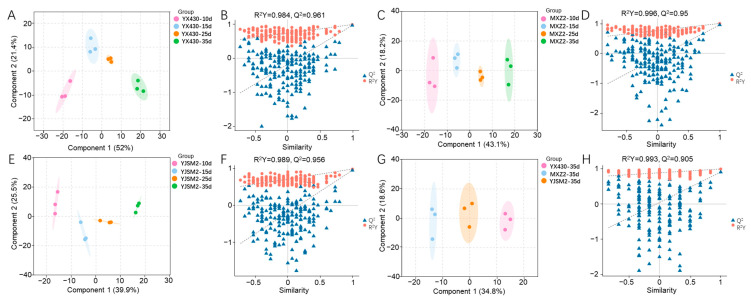
OPLS-DA score plots of YX430 (**A**), MXZ2 (**C**), and YJSM2 (**E**). Two hundred permutation tests of YX430 (**B**), MXZ2 (**D**), and YJSM2 (**F**). (**G**) OPLS-DA score plots of brown rice in 30 d. (**H**) Two hundred permutation tests of brown rice in 30 d.

The supervised OPLS-DA model is effective in filtering out orthogonal variables that are irrelevant to the classification variables in metabolites, thereby distinguishing observations between groups and identifying differential metabolites [38]. We employed OPLS-DA to analyze all VOCs of YX430, MXZ2, and YJSM2, respectively (Figure 2A,C,E), and brown rice in 30 d (Figure 2G). The accuracy of the model was validated through 200 permutation tests, resulting in R2Y and Q2 scores exceeding 0.9 (Figure 2B,D,F,H), indicating that the OPLS-DA model did not suffer from overfitting [39]. Each stage of the three varieties can be independently modeled into one group. At maturity, YX430, MX2, and YJSM2 were modeled as one group, respectively.

### 3.3. Analysis of Differential VOCs at Various Ripeness Levels of Rice Grains

**Figure 3:**
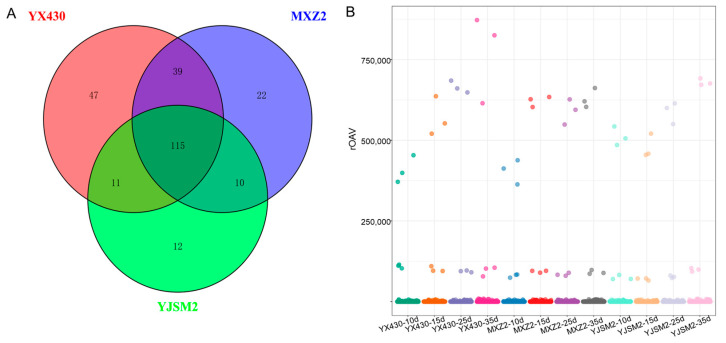
(**A**) Venn diagram of different VOCs among YX430, MXZ2, and YJSM2 during grain ripening. Red means YX430, blue means MXZ2, and green means YJSM2. (**B**) Scatterplot of rOAV odor activity values. The horizontal coordinate represents different groups, and the vertical coordinate represents the rOAV value of VOCs. These points represent all VOCs.

The value of variable importance in projection (VIP) reflects the contribution of each VOC to groups. Based on the VIP values from the OPLS-DA model, we screened for significant variances using the following criteria: VIP ≥ 1 and *p*-value < 0.05. This analysis identified 212, 186, and 148 significantly different compounds in the maturation of YX430, MXZ2, and YJSM2 cultivars, respectively (Tables S2–S4). It is mainly composed of ester, heterocyclic compounds, hydrocarbons, and terpenoids. The variety of these substances was abundant in the metabolic profile we examined. A Venn diagram was made to illustrate the shared differential VOCs among YX430, MXZ2, and YJSM2 during grain ripening (Figure 3). Across three pairwise comparisons, 115 overlapping differential VOCs were identified as key markers for rice grains (Tables S5), including esters (**23**), heterocyclic compounds (**21**), terpenoids (**16**), hydrocarbons (**10**), alcohols (**10**), ketones (**9**), aldehydes (**8**), aromatics (**7**), acids (**3**), amines (**2**), phenols (**1**), nitrogen compounds (**2**), sulfur compounds (**1**), and other compounds (**1**). These substances may be used as markers for different ripening stages of rice grains.

Additionally, a total of 39 differential VOCs were altered exclusively in YX430 and MXZ2, but not in YJSM2. Among these, 24 VOCs were annotated as contributing to aroma (Table S6). These include five heterocyclic compounds (succinimide, 3-acetyl-2,5-dimethylfuran, 2-hexylthiophene, 5-ethyl-3-hydroxy-4-methyl-2(5H)-furanone, and 2-isobutylthiazole), five esters (butyl hexanoate, 2-methylbutyl hexanoate, ethyl 2-octenoate, ethyl sorbate, and 2-methylbutyl 2-methylpropanoate), five terpenoids (Para-menthatriene, carvone, 2,6,6-trimethyl-2,4-cycloheptadien-1-one, L-menthol, and geraniol), four aldehydes (hexanal, cis-3-hexenal, nonanal, and (E)-cinnamaldehyde), three aromatics (4-allylphenol, o-xylene, and styrene), one nitrogen compound (2-nonenonitrile), and one sulfur compound (3-(methylthio)propanoic acid). These compounds may serve as markers to distinguish between aromatic and non-aromatic rice grains at different ripening stages.

### 3.4. Differential VOCs’ Analysis in Aromatic and Non-Aromatic Brown Rice

To analyze the differential VOCs between aromatic (YX430 and MXZ2) and non-accented (YJSM2) rice at maturity (35 d), we used VIP > 1, *p* < 0.05, and |log2FC| > 1 for two-group analysis as screening criteria. As illustrated in Table 1, the differential VOCs varied significantly among the comparisons. Specifically, 19 significantly different metabolites were identified between MXZ2 and YX430 (5 upregulated and 14 downregulated), 12 between YJSM2 and YX430 (0 upregulated and 10 downregulated), and 23 between YJSM2 and MXZ2 (12 upregulated and 15 downregulated). The close genetic background shared by YX430, MXZ2, and YJSM2 may account for the observed differences in VOCs in these pairwise comparisons. Among the upregulated metabolites in aromatic rice compared to YJSM2, eight were identified, five of which are associated with specific flavors: α-calacorene (woody), calamenene (herbal, spicy), 2-acetyl-1-pyrroline (popcorn, toasted, and grain), 1-nonanol (fresh, floral, and rose), and heptanal (fresh, fatty, and green). α-Calacorene and calamenene are the terpenoid organic compound, which are often found in Chinese medicinal materials. A study indicated that the N-heterocyclic class is the major distinguishing class between scented and non-scented rice [4]. As previously reported [2], 2-acetyl-1-pyrroline is a typical heterocyclic compound that distinguishes aromatic and non-aromatic rice. This study has identified, for the first time, the differing presence of 1-nonanol and heptanal in both aromatic and non-aromatic rice. These substances may serve as marker VOCs that distinguish aromatic from non-aromatic rice.

### 3.5. Odor Profiles of Grain During the Ripening of Rice

The contribution of each VOC to the overall aroma of rice is influenced not only by its concentration but also its odor threshold. Volatile compounds with an rOAV ≥ 1 are considered to contribute significantly to the overall flavor of rice grains [32]. To identify the key aroma-active compounds in rice grains at various ripening stages, we performed rOAV analyses on all detected VOCs. In total, 65 VOCs with rOAV above 1 were identified as key aromatic compounds in rice grains (Figure 3B), with their threshold values and odor descriptions listed in Table S7. Among these, 7 aroma components had rOAV greater than 1000, 13 had rOAVs between 100 and 1000, and 36 had rOAV between 1 and 100, detected across the four ripening stages. Additionally, nine aroma components with rOAV greater than 1 were detected only at one or two ripening stages. These findings suggest that these 65 substances play a crucial role in contributing to the flavor profile of rice grains.

Combining the differential metabolite analyses in seeds of different maturity levels (Tables S5 and S7), we identified 25 VOCs (with rOAV > 1) that varied significantly with grain maturation and contributed to fragrance in all three rice varieties. Except for methyl salicylate, the remaining substances accumulated in large quantities as the grains matured. Notably, (Z)-6-nonenal, (Z,Z)-3,6-nonadienal, and 2-thiophenemethanethiol had rOAVs exceeding 1000 at all four stages of grain development. Additionally, 5-methyl-2-furanmethanethiol, 2-methylisoborneol, 3-mercaptohexyl acetate, 2,2,6-trimethyl-cyclohexanone, and 3-octen-2-one had rOAVs greater than 100 at all stages, reaching their maximum levels by 35 days. The accumulation of 3-octen-2-one recorded in mature grains aligns with previous reports [4]. Moreover, other compounds, including 1-(2-thienyl)-ethanone, (Z)-3-hexen-1-ol, 2-acetyl-1-pyrroline, 2-furanmethanol, benzyl alcohol, (Z)-3,7-dimethyl-1,3,6-octatrien, 2-methylnaphthalen, naphthalene, (-)-perillaldehyde, (Z)-2-decenal, (E)-4-decenal, propanoic acid hexyl ester, and heptanoic acid ethyl ester, also played important roles in aroma formation during seed ripening despite having rOAVs of less than 100. However, p-cresyl acetate and n-butyl butanoate contributed very little to the overall flavor. The content of 2-AP increased with the ripening of rice grains, consistent with the finding that a previous publication reported [4]. (Z)-3-Hexen-1-ol, a green, grass-flavored compound found in many fresh fruits and vegetables, was present in rice [40]. Benzyl alcohol was more abundant in aromatic rice than in non-aromatic rice, contributing a slightly sweet flavor [41,42]. Based on the ratio of content at 35 days to 10 days, exceeding 2, and the rOAV results (Table S7), the compounds (Z)-6-nonenal, (Z,Z)-3,6-nonadienal, 2-thiophenemethanethio, 5-methyl-2-furanmethanethiol, 2,2,6-trimethyl-cyclohexanone, and 3-octen-2-one were the most variable, and they contributed significantly to the flavor during rice-grain maturation. A rapid increase in the content of these six metabolites indicates grain maturity, making them potential markers for identifying ripening stages in rice grains.

Combining the results of the differential metabolite analysis between aromatic and non-aromatic brown rice (Table 1 and Table S7) identified three metabolites with an rOAV > 1: 2-AP, heptanal, and 1-nonanol. The levels of these compounds were higher in aromatic rice (YX430 and MXZ2) compared to non-aromatic rice (YJSM2), contributing solely to the aroma of aromatic brown rice. Notably, 2-AP has long been recognized as a key marker for distinguishing between aromatic and non-aromatic rice. The 2-AP content and rOAV in ripening aromatic rice grains were the highest, but they made no significant contribution to the fragrance of non-aromatic rice. Heptanal significantly contributed to the aroma of aromatic rice in all four ripening stages, but it exhibited only a weak contribution to non-aromatic brown rice. Heptanal had higher content in cooked rice [17]. 1-Nonanol made a significant contribution to the aroma of brown rice, especially aromatic rice. It was also found in wild rice, providing floral and grassy aromas [43]. Therefore, 2-AP, heptanal, and 1-nonanol are key-marker metabolites that differentiate aromatic from non-aromatic brown rice.

The remaining 37 VOCs (Table S7), which also varied by grain maturity but did not significantly differ between aromatic and non-aromatic varieties, form the fundamental VOC profile contributing to the natural aroma of rice. Among these, 5-ethyl-3-hydroxy-4-methyl-2(5H)-furanone and 2-methyltetrahydrofuran-3-one had the highest rOAVs across all four stages, imparting green-fruit, sweet, maple-sugar, and nutty notes, which are characteristic of food flavors [44]. Aldehydes are among the most important volatiles in rice; a total of 15 aldehydes contribute to the flavor of rice. Previous research has indicated that hexanal, pentanal, and (E)-2-octenal are the primary compounds responsible for the flavor differences between aromatic and non-aromatic rice [6,8]. Furthermore, the hexanal content in ground rice is higher that than in whole rice [45]. Other VOCs, such as heterocyclic compounds, esters, alcohols, terpenoids, and ketone, also affect the aroma of rice. The heterocyclic compound 2-pentylfuran, which imparts a strong nutty and bitter-like aroma, has also been identified in rice and cooked rice [17,46]. It is produced from an oxidation product of methyl linoleate [47]. Alcohol contributed greatly to the odor of rice. 1-Octen-3-ol, known for its raw mushroom-like odor, was found to decrease in content as grains matured [34]. 1-Heptanol provides a sweet and nutty taste only in ripening grains. The rOAV of linalool ranges from 15.51 to 20.4 in brown rice, and linalool has also been detected in rice bran [48], suggesting that it may be a major component of brown rice flavor. Overall, most VOCs in aromatic and non-aromatic rice were the same, except for some special substances.

## 4. Conclusions

This investigation has provided a detailed flavor-profile analysis of rice-grain samples from three varieties (the aromatic rice varieties YX430 and MXZ2, and the non-aromatic variety YJSM2) at four critical ripening stages using HS-SPME-GC-MS, detecting a total of 417 VOCs. PCA and HCA demonstrated that VOC profiles from different stages were well separated and easily distinguishable. According to OPLS-DA and Venn analysis, 115 VOCs were identified as differential metabolites across the four ripening stages in the three cultivars. In addition, 20, 13, and 23 significantly different VOCs were identified between the MXZ2-vs.-YX430, YJSM2-vs.-YX430, and YJSM2-vs.-MXZ2 comparisons, respectively. Through an rOAV analysis, 65 VOCs were identified as key aromatic compounds contributing to the aroma of rice. Most aroma-active compounds showed an accumulation pattern during grain ripening, contributing to green, sweet, fruity, nutty, popcorn, roasted, fatty, and minty odors and thereby enhancing the aroma of rice grains. The accumulation of (Z)-6-nonenal, (Z,Z)-3,6-nonadienal, 2-thiophenemethanethiol, 5-methyl-2-furanmethanethiol, 2,2,6-trimethyl-cyclohexanone, and 3-octen-2-one was indicative of rice-seed maturity. Additionally, 2-AP, heptanal, and 1-nonanol were identified as key-marker metabolites distinguishing aromatic from non-aromatic brown rice. These insights help improve the understanding of flavor dynamics in rice obtained from different ripening stages, and they provide a theoretical basis for the comprehensive improvement of aromatic rice varieties.

## Figures and Tables

**Table 1:** The VOCs that serve as marker compounds to distinguish between YX430, MXZ2, and YJSM2 at the ripening stage.

Compounds	Log2FC			Odor
	MXZ2 vs. YX430	YJSM2 vs. YX430	YJSM2 vs. MXZ2	
Terpenoids				
β-Cubebene	1.41	ns	−2.39	Citrus, fruity, radish
Para-menth-3-en-1-ol	1.28	ns	−3.24	Dry, woody, musty
Isoledene	1.08	−1.16	−2.24	-
Cis-p-menthan-1-ol	1.06	−1.00	−2.06	-
α-Calacorene	ns	−1.72	−2.50	Woody
Calamenene	ns	−2.30	−2.88	Herbal, spice
(8R,8aS)-8,8a-Dimethyl-2-(propan-2-ylidene)-1,2,3,7,8,8a-hexahydronaphthalene	ns	−2.37	−3.02	-
Cis-Cyclohexanol, 1-methyl-4-(1-methylethyl)	ns	ns	−1.54	-
Hydrocarbons				
4-Methyltetradecane	−1.62	ns	1.38	-
Tetradecane	−1.79	ns	1.12	Mild, waxy
Pentadecane	−1.57	ns	1.32	Waxy
Hexadecane	−1.50	ns	1.12	Alkane
(E)-2-Tetradecene	−1.52	ns	1.13	-
Tetradecane, 2-methyl-	−1.55	ns	1.18	-
Bicyclo(3.3.1)non-2-ene	ns	−1.17	ns	-
Heterocyclic compound				
Platynecin	−1.22	ns	1.05	-
5-Aminotetrazole	−1.46	ns	1.23	-
2-Acetyl-1-pyrroline	ns	−3.68	−3.39	Popcorn, toasted, grain
1,5,6-Trimethyl-azacyclohexan-3-one	ns	ns	−1.68	-
6-Methyl-6-(5-methylfuran-2-yl)heptan-2-one	−2.33	ns	2.03	-
4-Acetyl-pyrazol	ns	ns	−1.20	-
1,2,4,5-Tetrazin-3-Amine	ns	ns	−1.23	-
Ketone				
Tetrahydrogeranylacetone	−1.60	ns	ns	Dry, musty
2-keto-1,1,10-trimethyl-Δ8-octalin	−1.65	ns	1.53	-
Dihydro-beta-ionone	−2.73	ns	2.58	Earthy, woody
Ester-				
δ-Decalactone	−1.82	ns	1.41	Creamy, coconut, fruity
Alcohol				
1-Nonanol	ns	−1.60	−1.01	Fresh, floral, rose
2-Octanol	1.01	ns	−1.09	Fresh, herbal, earthy
Acid				
Undecylenic acid	−1.63	ns	ns	Sweet, woody
Compounds	Log2FC			Odor
	MXZ2 vs. YX430	YJSM2 vs. YX430	YJSM2 vs. MXZ2	
Aldehyde				
Heptanal	ns	−2.55	−2.45	Fresh, fatty, green,
Cyclohexanecarboxaldehyde	ns	−1.07	ns	

Log2FC is the logarithmic value of the multiple of the change in content (µg/g) in the treatment group relative to the control group. MXZ2 vs. YX430, VOCs’ content difference in grains between treatment group MXZ2 and control group YX430; YJSM2 vs. YX430, VOCs’ content difference in grains between treatment group YJSM2 and control group YX430; YJSM2 vs. MXZ2, VOCs’ content difference in grains between treatment group YJSM2 and control group MXZ2. ns: There was no significant difference between groups; “-” means no finding.

## Data Availability

The original contributions presented in this study are included in the article/Supplementary Materials. Further inquiries can be directed to the corresponding author.

## Supplementary Materials

The following supporting information can be downloaded at https://www.mdpi.com/article/10.3390/foods13233776/s1: Figure S1. Total ion diagram for mixed samples spectrum; Figure S2. The loading plot of the PCA; Table S1. 417 VOCs identified by HS-SPME-GC–MS; Table S2. 212 metabolic differentials were obtained from the metabolites of YX430 grains by limiting the conditions to VIP > 1 and *p* < 0.05; Table S3. 187 metabolic differentials were obtained from the metabolites of MXZ2 grains by limiting the conditions to VIP > 1 and *p* < 0.05; Table S4:148 metabolic differentials were obtained from the metabolites of YJSM2 grains by limiting the conditions to VIP > 1 and *p* < 0.05; Table S5. A total of 115 differential VOCs in the ripening of rice grains of YX430, MXZ2, and YJSM2; Table S6. A total of 39 differential VOCs in the ripening of rice grains of YX430 and MXZ2, but no difference in YJSM2; Table S7. The volatile compounds with OAV values above 1 in rice grains produced from YX430, MXZ2, and YJSM2.
