# Supplementary figures and images for: Analysis of Volatile Compounds’ Changes in Rice Grain at Different Ripening Stages via HS-SPME-GC–MS
Source: Foods. 2024 Nov 25;13(23):3776. doi: 10.3390/foods13233776 (PMC11640236; doi:10.3390/foods13233776)

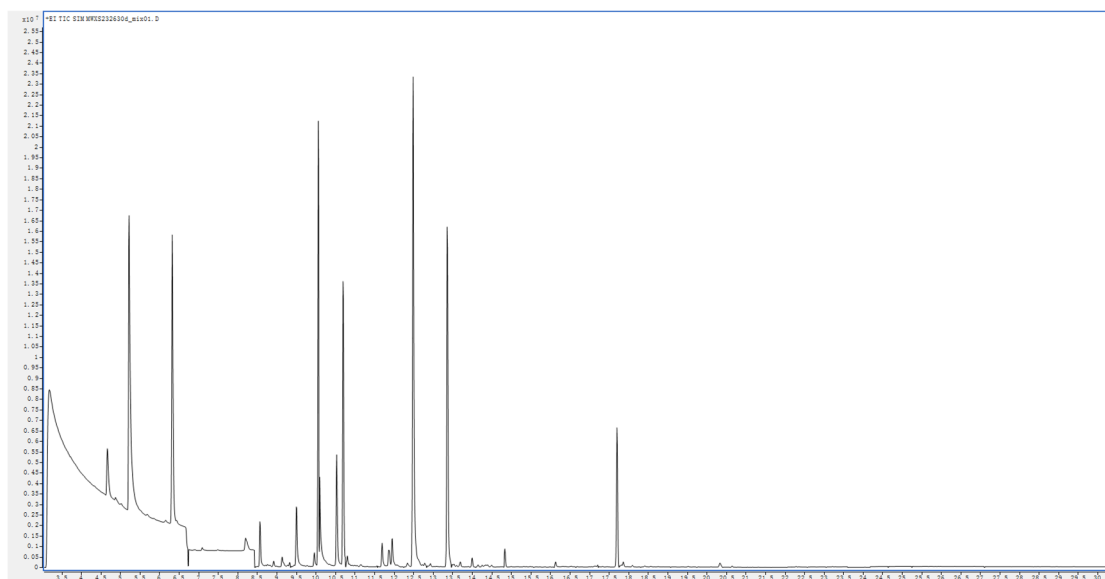

**Figure S1:** Total ion diagram for mixed samples spectrum

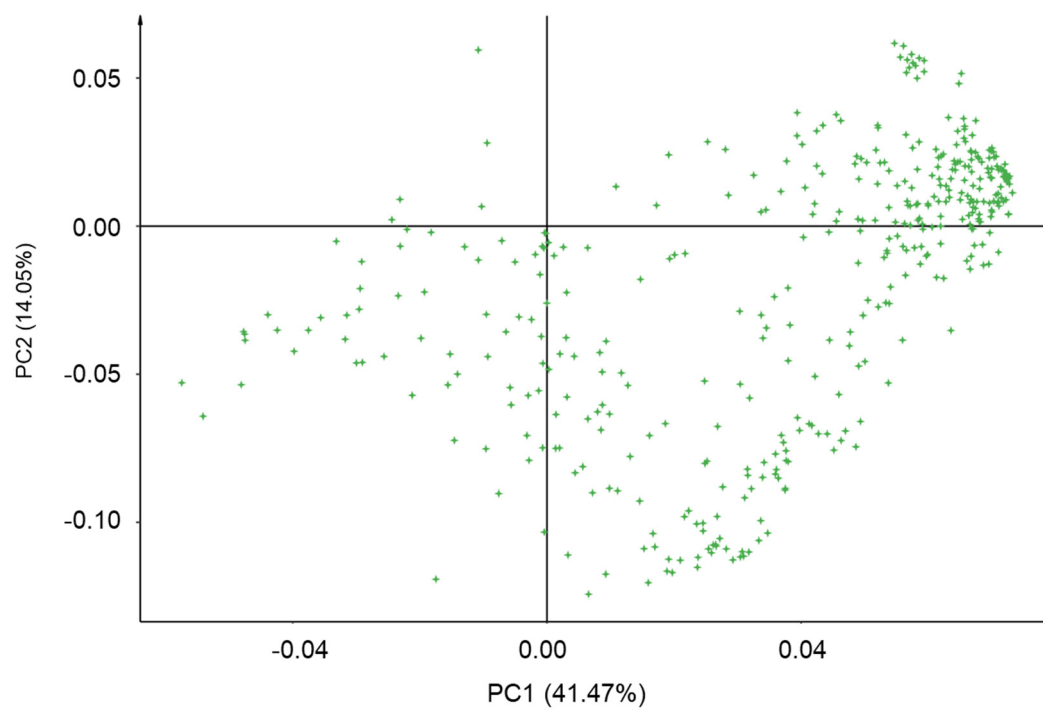

**Figure S2:** The loading plot of the PCA

Supplement: Supplementary file 1 [file foods-13-03776-s001.zip › Supplementary figures.pdf]
